# Electron beam energy QA — a note on measurement tolerances

**DOI:** 10.1120/jacmp.v17i2.6049

**Published:** 2016-03-08

**Authors:** Juergen Meyer, Matthew J. Nyflot, Wade P. Smith, Landon S. Wootton, Lori Young, Fei Yang, Minsun Kim, Kristi R. G. Hendrickson, Eric Ford, Alan M. Kalet, Ning Cao, Claire Dempsey, George A. Sandison

**Affiliations:** ^1^ Department of Radiation Oncology University of Washington Seattle WA USA

**Keywords:** TG‐142, linac electron beam energy check, monthly QA, constancy check, TG‐198

## Abstract

Monthly QA is recommended to verify the constancy of high‐energy electron beams generated for clinical use by linear accelerators. The tolerances are defined as 2%/2 mm in beam penetration according to AAPM task group report 142. The practical implementation is typically achieved by measuring the ratio of readings at two different depths, preferably near the depth of maximum dose and at the depth corresponding to half the dose maximum. Based on beam commissioning data, we show that the relationship between the ranges of energy ratios for different electron energies is highly nonlinear. We provide a formalism that translates measurement deviations in the reference ratios into change in beam penetration for electron energies for six Elekta (6‐18 MeV) and eight Varian (6‐22 MeV) electron beams. Experimental checks were conducted for each Elekta energy to compare calculated values with measurements, and it was shown that they are in agreement. For example, for a 6 MeV beam a deviation in the measured ionization ratio of ±15% might still be acceptable (i.e., be within ±2 mm), whereas for an 18 MeV beam the corresponding tolerance might be ±6%. These values strongly depend on the initial ratio chosen. In summary, the relationship between differences of the ionization ratio and the corresponding beam energy are derived. The findings can be translated into acceptable tolerance values for monthly QA of electron beam energies.

PACS number(s): 87.55, 87.56

## I. INTRODUCTION

Recommendations for quality assurance (QA) of clinical medical linear accelerators (linacs) are provided by the AAPM task group 142 (TG‐142) report,^(1)^ which updates and in part supersedes the AAPM TG‐40 report.^(2)^ The recommended QA tests are separated into daily, monthly, and annual QA, and also incorporate recommendations for the latest linac technologies and contemporary treatment techniques. The premise is to monitor on a regular basis deviations from baseline values acquired during commissioning of the linac. TG‐142 specifies recommendations for the frequency of the test and acceptable tolerance values that ensure safe and accurate clinical operations. No recommendations are made to specify how the QA test should be performed and what equipment or software should be used for analysis. Due to the complexity of the TG‐142 report, TG‐198 was formed to provide specific procedural guidelines for performing the recommended TG‐142 tests. The report, which is scheduled for release at the end of 2016, is widely anticipated by the medical physics community.

The focus of this article is on the recommended linac monthly QA test, Electron Beam Energy Constancy. The incident energy spectrum of an electron beam is proportional to its penetrating power, but this spectrum is not easily measurable in clinical practice. Therefore, for QA purposes, the $R_{50}$ depth at which the percentage depth dose (PDD) curve corresponds to 50% of the maximum absorbed dose is instead typically used to characterize the penetration of clinically used electron beams. For more detailed analysis of the complex energy spectrum several parameters may further be used to describe the beam energy at the phantom surface, such as the most probable energy, $E_{p,0}$, and the mean energy, $E_{0}$. The beam quality index relating to $R_{50}$ in electron beam dosimetry is generally used and, as such, is defined in, for example, the dosimetry protocols TG‐51^(3)^ and TRS‐398.^(4)^

With regard to clinical practice, the implementation of a representative beam quality check for monthly QA is typically performed using a source‐to‐surface (SSD) setup of 100 cm and standard reference field size of $10\times 10\;{\text{cm}}^{2}$. Measurements are made at or near the depth of dose maximum, $d_{\text{max}}$, and at or near the depth of half the dose maximum for the particular electron energy under consideration. For routine monthly QA a Solid Water phantom is commonly used as the absorption medium, with various water‐equivalent materials available that mimic water such as polystyrene, polymethylmethacrylate (PMMA), A‐150 tissue equivalent plastic,^(5)^ Solid Water WT1 (Gammex RMI, Middleton, WI), Solid Water RMI‐457 (Gammex), Plastic Water (Computerized Imaging Reference Systems Inc., Norfolk, VA), or Virtual Water (Standard Imaging Inc., Middleton, WI).^(6)^ The effects of phantom material on electron beams are discussed elsewhere.^(7)^ Common dosimeters used for the energy check are ion chambers, either a parallel plate or cylindrical Farmer type. Strictly speaking, ion chambers measure depth ionization for electron beams and, in order to convert to dose as is required for $R_{50}$, the mass collision stopping power ratios water to air need to be applied, plus factors that take into account electron fluence perturbations. However, one needs to bear in mind that, while exact determination of $R_{50}$ is required for dosimetry protocols such as TG‐51, the beam quality index for monthly QA is merely a constancy test that does not require exact determination of $R_{50}$. Different methods have been proposed to determine energy constancy for QA checks.^8^, ^9^, ^10^, ^11^, ^12^, ^13^, ^14^ A common method is to take the ratio of ion chamber measurements at two depths. This energy check reference value is henceforth referred to as the ratio. Typically this will result in a ratio close to 50%. This is in contrast to TG‐24 and TG‐40 where the specifications were near $R_{80}$. For convenience it is common in clinical practice to group the measurement depths for different electron energies together, which can result in ratios that may range between 30% and 70%. This is deemed acceptable as long as the ratios remain constant from month to month.

The difficulty arises when relating the tolerances given in TG‐142 to acceptable measurement deviations. It is not straightforward to relate and interpret what measurement error relative to the baseline energy ratio is acceptable.^(15)^ For clinical practice the question is whether a measured shift in energy is within the ±2 mm tolerance specified in TG‐40 and TG‐142. For completeness it should be noted that TG‐40 specifies an “Electron central axis dosimetry parameter constancy (PDD)” tolerance of “2 mm at therapeutic depth,” while in an older task group report (TG‐24) from 1994, electron beam energy constancy was recommended to be verified at a depth of 80% dose with a tolerance of ±3 mm.^(16)^ TG‐142 does not specify a depth of interest and also specifies a tolerance of “2%” without further details as to what exactly it refers to. Our experience has shown that variations of >±2 mm in the beam penetration are possible. The available data in the literature about the long‐term stability of electron energies of modern linacs is relatively sparse,^(17)^ and mainly focus on the stability of dedicated accelerators like the intraoperative Mobetron (IntraOp Medical Corporation, Sunnyvale, CA).^18^, ^19^

The aim of this work is to relate, independent of dosimeter and phantom, the measurement tolerances for a range of ionization ratios to changes in penetrative power for the most common electron energies. None of the above‐referenced methods for energy checks address this issue.

## II. MATERIALS AND METHODS

**Figure 1:**
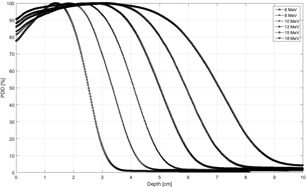
Reference data for Elekta electron PDDs from commissioning used for mapping out ionization ratios.

To keep this work independent of detector and solid water material, and because QA tolerances should approximately be independent of using ionization or dose, previously measured electron commissioning PDD data were used henceforth. MATLAB (MathWorks, Natick, MA) code was written to generate ratios from the PDD data for electron energies 6 MeV, 8 MeV, 10 MeV, 12 MeV, 15 MeV, and 18 MeV. The data were obtained from a recently commissioned Elekta (Elekta Inc., Atlanta, GA) Infinity linac with Agility head and the standard $10\times 10\;{\text{cm}}^{2}$ electron applicator. Commissioning data were acquired in OmniPro Accept (IBA Dosimetry GmbH, Schwarzenbruck, Germany) with an IBA Electron Field Detector 3G and Reference Dosimetry Diode 3G in continuous ratio acquisition mode. Measurements were made in water with an IBA Blue Phantom and CU500E controller. PDD data were processed in OmniPro Accept with linear interpolation to 0.2 mm and smoothed with an arithmetic mean filter over 4 mm. The beams were commissioned according to the $R_{50}$ values recommended by the manufacturer (i.e., the penetration to which the beams were tuned at the factory prior to installation of the linac). For reference, the PDDs are shown in Fig. 1, and the corresponding parameters describing the beams are given in Table 1. For each ratio the depth at dose maximum was kept constant, but the second depth was varied by ±1 mm and ±2 mm in order to map out its impact on the initial ratio. This assumes that the shape of the distal falloff of the PDD curves does not vary markedly for small changes in energy consistent with ±2 mm in $R_{50}$. The same analysis was also carried out for the golden beam data (GBD) for Varian linacs (Varian Medical Systems, Paolo Alto, CA).

For illustrative purposes the range of ratios was plotted from 10% to 90%, although in clinical practice it is not recommended to deviate from 50% by such a large amount. To test the sensitivity of a monthly two‐depth energy constancy check, the relative difference in the ionization ratio was measured on the Elekta linac for each energy with an Exradin A19 ion chamber (Standard Imaging) in solid water (Gammex) using a $10\times 10\;{\text{cm}}^{2}$ field at 100 SSD.

**Table 1:** Electron beam data specifications. Values are for Elekta electron energies. The measured ratios were obtained by dividing the measured values at $R_{50}$ and $d_{\text{max}}$

*Energy (MeV)*	*6*	*8*	*10*	*12*	*15*	*18*
Most Probable Energy, $E_{p,0}$	6.4	8.6	10.2	12.4	14.6	17.5
Mean Energy, $E_{0}$	5.9	7.8	9.6	11.7	13.8	16.5
$D_{\text{max}}$ (cm)	1.4	1.9	2.3	2.7	3.1	2.9
$R_{50}$ (cm)	2.53	3.36	4.10	5.01	5.93	7.08
Measured Ratio	54%	50%	52%	58%	54%	51%

This setup was chosen as representative of a typical monthly electron energy check. Solid water was stacked to position the chamber at approximately the depth of $d_{\text{max}}$, and then $R_{50}\pm 0,1$, and 2 mm. At each depth, three irradiations were performed and the average chamber response was recorded for a total of 90 measurements. The measurements centered around $R_{50}$ were then normalized to $d_{\text{max}}$, and the difference in response from $R_{50}$ was calculated.

## III. RESULTS

**Figure 2:**
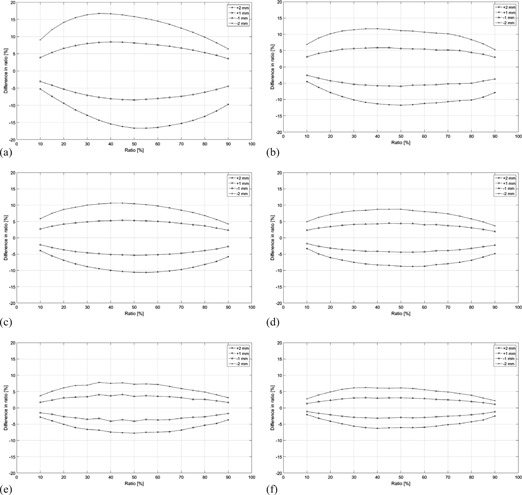
Ionization ratios as a function of ±1 and ±2 mm shifts in beam penetration for Elekta beams with nominal energies (a) 6 MeV, (b) 8 MeV, (c) 10 MeV, (d) 12 MeV, (e) 15 MeV, and (f) 18 MeV. The y‐axis represents the difference between a given ratio and the resulting ratio if the beam penetration has changed.

The results of the tolerances for the ionization ratios between 10% and 90% are given in Figs. 2(a) to 2(f) for the Elekta commissioning data. The tolerances, defined as the difference between a reference ratio and the resulting measured ionization ratio if the beam penetration has changed, are plotted for shifts in beam energy of +2 mm,+1 mm,−1 mm, and −2 mm. As an example for an initial reference ratio of 50% for the 6 MeV electrons, ratios of 33%, 42%, 58%, and 66% would be obtained if the beam penetration had changed by +2 mm,+1 mm,−1 mm, and −2 mm, respectively. The differences of −17%,−8%,8%, and 16%, respectively, are plotted in Fig. 2(a) for the initial reference ratio of 50%.

In general, the closer the ratio is to 50%, the larger the acceptable measurement deviations are. For smaller and larger ratios the acceptable deviations become smaller, following a nonlinear trend. The tolerances corresponding to beam penetration shifts in positive and negative directions are nonsymmetric. The higher the energy, the smaller the tolerances. The differences plotted on the y‐axes in Fig. 2 correspond to the measured deviation if a beam energy shift was present.

**Figure 3:**
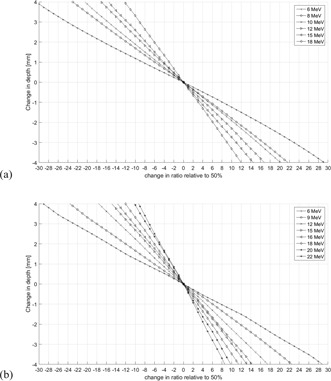
Conversion between changes in ratio and the corresponding change in beam penetration. (a) For Elekta and (b) Varian linac. It is assumed that the change is relative to a reference ratio of 50%.

In Fig. 3 similar information is presented for both Elekta and Varian data but in a format that directly converts relative changes in the ratio to changes in penetration. Here it was assumed that the reference ratio was 50% and the simulated changes are relative to 50%. For the example given above, the same tolerance values are obtained by following a given shift on the y‐axis in horizontal direction until it intercepts the graph for a given energy. The numerical values for a range of ratios are given in Table 2 for the Elekta and Table 3 for the Varian data.

**Figure 4:**
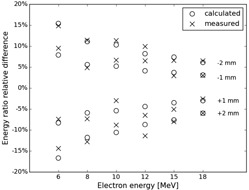
Measurement results showing the relative difference in the ratio for measurements at ±1 and ±2 mm from the nominal $R_{50}$ depth. The expected differences are plotted as a circle and the measured values are indicated by a cross.

The measured values compared to the calculated ones are plotted in Fig. 4. The calculated values are based on the measured ratios shown at the bottom of Table 1. The measured ratios agree well with the calculated ones with a maximum difference of about 3%.

**Table 2:** Numerical tolerance values for different beam energies for Elekta linac. The values represent the relative difference in the ratio in percentage for ±1 and 2 mm shifts in beam energy.

*Energy (MeV)*	*6*				*8*				*10*			
*Distance (mm)*	−2	−1	*1*	*2*	−2	−1	*1*	*2*	−2	−1	*1*	*2*
30%	16.4	8.0	−7.1	−12.9	11.4	5.6	−5.4	−10.1	10.0	4.8	−4.6	−8.8
40%	16.6	8.4	−8.1	−15.4	11.7	5.9	−5.8	−11.3	10.6	5.2	−5.1	−10.0
Ratio 50%^a^	15.8	8.1	−8.4	−16.7	11.1	5.6	−5.9	−11.8	10.4	5.3	−5.3	−10.6
60%	14.5	7.6	−8.0	−16.5	10.6	5.5	−5.6	−11.2	9.7	5.0	−5.2	−10.5
70%	12.5	6.7	−7.4	−15.3	10.2	5.2	−5.2	−10.7	8.4	4.4	−4.7	−9.7
*Energy (MeV)*	*12*				*15*				*18*			
*Distance (mm)*	−2	−1	*1*	*2*	−2	−1	*1*	*2*	−2	−1	*1*	*2*
30%	8.3	4.1	−3.8	−7.5	7.0	3.4	−3.5	−6.6	6.1	3.0	−2.9	−5.6
40%	8.7	4.3	−4.2	−8.3	7.5	3.7	−4.1	−7.5	6.1	3.0	−3.2	−6.3
Ratio 50%^a^	8.7	4.3	−4.4	−8.7	7.3	3.5	−4.1	−7.8	6.1	3.1	−2.9	−6.1
60%	8.0	4.0	−4.3	−8.7	7.2	3.5	−3.7	−7.4	5.5	2.8	−3.0	−6.0
70%	7.3	3.7	−3.9	−8.0	5.9	3.1	−3.2	−6.8	4.9	2.5	−2.6	−5.2

a ^a^ Ratios in this row correspond to the data plotted in Fig. 3.

**Table 3:** Numerical tolerance values for different beam energies for Varian linac. The values represent the relative difference in the ratio in percentage for ±1 and 2 mm shifts in beam energy

*Energy (MeV)*	*6*				*9*				*12*				*15*			
*Distance (mm)*	−2	−1	*1*	*2*	−2	−1	*1*	*2*	−2	−1	*1*	*2*	−2	−1	*1*	*2*
30%	15.1	7.1	−7.0	−12.7	12.0	6.1	−5.3	−10.2	8.6	4.2	−4.1	−8.0	6.9	3.3	−3.5	−6.7
40%	15.7	8.2	−7.2	−14.6	12.1	6.0	−6.0	−11.8	8.8	4.2	−4.4	−8.7	7.4	3.7	−3.8	−7.3
Ratio 50%^a^	15.4	7.8	−7.9	−15.5	11.8	6.1	−6.1	−12.0	9.0	4.7	−4.7	−9.0	7.2	3.8	−3.8	−7.4
60%	14.3	7.2	−8.0	−15.7	11.0	5.7	−5.7	−11.9	8.5	4.3	−4.3	−8.9	6.6	3.6	−3.5	−7.0
70%	12.6	7.0	−6.8	−14.9	9.6	5.0	−5.4	−11.1	7.4	3.8	−4.0	−8.3	5.9	3.1	−3.1	−6.1
*Energy (MeV)*	*16*				*18*				*20*				*22*			
*Distance (mm)*	−2	−1	*1*	*2*	−2	−1	*1*	*2*	−2	−1	*1*	*2*	−2	−1	*1*	*2*
30%	6.6	3.3	−3.1	−6.0	5.4	2.5	−2.8	−5.2	5.2	2.6	−2.5	−4.7	4.5	2.2	−2.1	−4.1
40%	7.0	3.5	−3.4	−6.7	6.0	3.0	−2.9	−5.8	5.1	2.5	−2.4	−5.0	4.4	2.2	−2.4	−4.6
Ratio 50%^a^	6.9	3.5	−3.3	−6.8	5.9	3.0	−3.1	−6.0	5.0	2.5	−2.5	−5.1	4.3	2.2	−2.4	−4.8
60%	6.2	3.1	−3.2	−6.6	5.2	2.7	−2.8	−5.7	4.5	2.3	−2.4	−4.8	4.1	2.1	−2.0	−4.3
70%	5.5	2.8	−3.0	−6.1	4.5	2.2	−2.5	−5.1	3.8	2.0	−1.9	−4.1	3.3	1.7	−1.8	−3.6

a ^a^ Ratios in this row correspond to the data plotted in Fig. 3.

## IV. DISCUSSION

The monthly energy constancy check for electron beams is an essential QA test that verifies that the beam energies are within acceptable clinical limits. While TG‐142 provides guidance on the acceptable change in penetration (i.e., ±2 mm/2%), it does not provide recommendations on how to translate measurement deviations for a ratio of measurements into a depth range. With regard to TG‐142, it is unclear to what the 2% tolerance corresponds. The formalism presented here maps out this relationship in general terms for a wide range of ratios for the most common clinical electron beams in the range 6‐18 MeV for Elekta and 6‐22 MeV for Varian beams. It can be seen that the tolerance is a nonlinear function of energy, the ratio chosen, and the direction of the beam energy shift.

For clinical routine it is important to be aware of this nonlinear behavior to avoid unnecessary adjustments to the electron beams when the deviation from the selected ratios appears large, while the beam is in fact still within the TG‐142 specifications. The nonlinear behavior is a function of the beam energy and hence the steepness of the descending PDD gradient. The approach presented here used the commissioning PDD data to map out the relationship. However, for QA checks Solid Water phantoms and ion chambers are typically used to perform these measurements. Heretofore, determination of tolerances often utilized measurements that added and removed 1‐2 mm layers of solid water to experimentally determine the tolerance doses that correspond to a ±1 mm and a ±2 mm shift in beam penetration due to changes in the beam energy. This ensures that appropriate tolerance values are used for a given setup in the clinic. Such measurements are not required using the methods and data presented in this work. Results for other Elekta or Varian beam energies could be interpolated from the data presented here. Results for other machines or energies might require applying the methods here for its PDD data. However, given the agreement of within 1% between the nominally identical beams (6, 12, and 18 MeV) between the two linac manufacturers investigated (see Tables 2 and 3), it is likely that interpolation will suffice.

Measurements were conducted to gauge the difference between the calculated tolerances based on commissioning data and actual measurements. The difference in ratios from the calculated 50% is given in Table 1. A range between 50% and 58% was obtained. This difference stems from several factors, including: 1) the fact that ion chambers measure depth ionization rather than dose, 2) the relatively large collecting volume of the Farmer‐type ion chamber, 3) measurement setup uncertainties, 4) the difference in electron stopping power between water and solid water, 5) the daily variation in the electron beam energy spectra,^(20)^ and 6) daily output fluctuations. It can be seen from Fig. 2 that the tolerances for the ratios for 50% and,

for example 58%, are very similar, which indicates that the calculated ranges and tolerances are reasonable despite the above‐mentioned differences between the intrinsic parameters for the calculated and the measured values. In Fig. 4, the direct comparison is shown between the calculated and measured ratios. All measurements follow the expected trend with the higher energies showing better agreement than the lower energies. This is not unexpected as the lower energies have a steeper dose gradient falloff than higher energies and are more susceptible to setup errors for both $d_{\text{max}}$ and $R_{50}$ measurements.

## V. CONCLUSIONS

We have shown that the tolerances for electron beam energy checks using the two‐depth method are highly nonlinear due to the differences in gradient of the PDD falloff region. Measurements confirmed this trend and were found to be in reasonable agreement. Figure 3 and Tables 2 and 3 show a direct conversion between the differences in measured ionization ratio and the corresponding change in beam penetration and hence beam energy. The approach is pragmatic, and we hope that this work will raise awareness and assist medical physicists in setting up tolerances for their own energy checks. We recommend that TG‐198 provide similar guidelines to ensure efficient QA procedures.

## COPYRIGHT

This work is licensed under a Creative Commons Attribution 4.0 International License.
